# High-Throughput Sequencing of Plasma MicroRNA in Chronic Fatigue Syndrome/Myalgic Encephalomyelitis

**DOI:** 10.1371/journal.pone.0102783

**Published:** 2014-09-19

**Authors:** Ekua W. Brenu, Kevin J. Ashton, Jana Batovska, Donald R. Staines, Sonya M. Marshall-Gradisnik

**Affiliations:** 1 School of Medical Science, Griffith Health Centre, Griffith University, Gold Coast, Queensland, Australia; 2 The National Centre for Neuroimmunology and Emerging Diseases, Griffith University, Gold Coast, Queensland, Australia; 3 Faculty of Health Sciences and Medicine, Bond University, Robina, Queensland, Australia; 4 Queensland Health, Gold Coast Public Health Unit, Robina, Gold Coast, Queensland, Australia; St. Georges University of London, United Kingdom

## Abstract

**Background:**

MicroRNAs (miRNAs) are known to regulate many biological processes and their dysregulation has been associated with a variety of diseases including Chronic Fatigue Syndrome/Myalgic Encephalomyelitis (CFS/ME). The recent discovery of stable and reproducible miRNA in plasma has raised the possibility that circulating miRNAs may serve as novel diagnostic markers. The objective of this study was to determine the role of plasma miRNA in CFS/ME.

**Results:**

Using Illumina high-throughput sequencing we identified 19 miRNAs that were differentially expressed in the plasma of CFS/ME patients in comparison to non-fatigued controls. Following RT-qPCR analysis, we were able to confirm the significant up-regulation of three miRNAs (*hsa-miR-127-3p, hsa-miR-142-5p* and *hsa-miR-143-3p*) in the CFS/ME patients.

**Conclusion:**

Our study is the first to identify circulating miRNAs from CFS/ME patients and also to confirm three differentially expressed circulating miRNAs in CFS/ME patients, providing a basis for further study to find useful CFS/ME biomarkers.

## Introduction

Chronic Fatigue Syndrome/Myalgic Encephalomyelitis (CFS/ME) is known to affect about 1–4% of individuals worldwide [1], [2]. CFS/ME is a multi-symptom disorder including profound disabling fatigue and post-exertional sickness, cognitive disturbances, tender or painful lymph nodes, muscle ache and pain and irregular sleep patterns [3]. There is evidence to suggest a dominant disruption of immunological process in CFS/ME and this may be characterised by reduced cytotoxic activity and increases in regulatory T cells [4], [5], [6]. In addition patients with CFS/ME may display differential expression in the mRNA and microRNA (miRNA) genes that regulate various physiological processes known to be dysregulated in CFS/ME including cytotoxicity, cytokine secretion and apoptosis [4], [7], [8], [9], [10], [11], [12]. Despite intensive research the pathophysiology of CFS/ME is not yet fully understood and clear diagnostic biomarkers remain elusive.

MicroRNAs are a class of small (typically 18–25 nucleotides in size) single-stranded, non-coding RNAs that regulate gene expression at the post-transcriptional level [13]. Their regulatory roles have been implicated in most biological processes including immunological, neurological and physiological processes [14], [15], [16], [17]. Differential expression of miRNA has been associated with over 300 diseases, including cancer, cardiomyopathies, neurological disorders and unexplained disorders such as CFS/ME [4], [18], [19], [20], [21]. Stable and reproducible extracellular miRNAs circulating in blood and present in other biofluids have recently been identified (16). It has been proposed that these miRNAs have the potential to be utilised as non-invasive novel biomarkers for disease diagnosis and prognosis [22], [23], [24], [25], [26].

In this study, we employed high-throughput sequencing (HTS) to globally profile circulating miRNA expression. This was followed by confirmative reverse transcription-quantitative PCR (RT-qPCR) to determine differential miRNA expression in CFS/ME.

## Materials and Methods

### Participants

CFS/ME patients (n = 20, age = 44.5±6.0 years) were recruited from a South-East Queensland patient database in Australia. Inclusion criteria for the CFS/ME participants were according to the American CDC 1994 case definition [26]. Non-fatigued controls (n = 20, age = 47.3±6.7 years) were recruited mainly from the general public, and were participants with no medical history or symptoms of persistent fatigue or illness. Individuals who were smokers, pregnant/breast-feeding or immobile were excluded from the study, as were individuals with autoimmune, thyroid or cardiac related disorders prior to the onset of CFS/ME. This project was approved by the Bond University Human Research Ethics Committee (BUHREC), approval number R0852A. All participants in the study provided informed and written consent prior to involvement in the study.

### Sample processing and RNA extraction

Plasma harvesting was performed immediately after peripheral blood collection. Briefly 10 mL of whole blood was collected from each participant into EDTA collection tubes. Plasma was immediately separated via centrifugation at 500×g for 10 min, and 5 mL of the plasma was transferred to a new tube and stored at -80°C. Prior to RNA extraction, the plasma samples were centrifuged for 10 min at 16,000×g at 4°C in order to remove any cellular debris left in the plasma. Circulating RNA was extracted from plasma using the miRNeasy Serum/Plasma kit (Qiagen, Hilden, Germany) according to manufacturer's instruction with minor modifications. Briefly, 1 mL of plasma was incubated with 5 mL of QIAzol Lysis Reagent for 10 min. Samples were processed using a vacuum manifold to help process the larger quantity of plasma. An additional 80% ethanol wash step was included and sample elution was performed twice using 15 µL of RNase-free water. The size, quantity and quality of the extracted circulating RNA was assessed using a small RNA chip on an Agilent 2100 Bioanalyzer (Agilent Technologies, Palo Alto, CA).

### MicroRNA profiling by HiSeq2000 sequencing

The six CFS/ME patients and six non-fatigued controls with the highest abundance of small RNA were used for HTS. Small RNA libraries were constructed using the TruSeq Small RNA Sample Preparation kit (Illumina, San Diego, CA) according to the manufacturer's protocols. Briefly, small RNA samples (5–10 ng) were ligated with 5′ and 3′ adapters, followed by reverse transcription-PCR (RT-PCR) for cDNA library construction and incorporation of index tags. The cDNA library fragments were purified separated on a 6% TBE PAGE gel and 145–160 bp size fraction containing miRNA inserts was isolated. The twelve cDNA library samples were pooled in equimolar amounts and used for cluster generation and sequence analysis in a single lane on an Illumina HiSeq2000 (50 bp single read). This work was performed at the Australian Genome Research Facility (AGRF).

### Sequencing data analysis

Raw FASTQ sequences were generated and demultiplexed using the Illumina CASAVA v1.8 pipeline. Per base sequence quality (quality score >30) was then assessed using the FastQC toolkit (http://www.bioinformatics.babraham.ac.uk/projects/fastqc). Prior to mapping the read data was pre-processed using the UEA small RNA Workbench (http://srna-workbench.cmp.uea.ac.uk) [27]. Briefly, the 3′ adapter sequences were trimmed, the read size filtered (16–35 nt), unique reads counted and low abundance reads (<10 reads) discarded. Unique sequence reads were then aligned to the human genome (hg18) and miRBase_v16 using the miRanalyzer web server tool (http://bioinfo2.ugr.es/miRanalyzer/miRanalyzer.php) [28]. Raw reads of the sequencing data are available at the NCBI Sequence Read Archive (SRA). Data are accessible through NCBI BioProject accession number PRJNA219428 at http://www.ncbi.nlm.nih.gov/bioproject/.

### Quantification of miRNA by RT-qPCR

The expression of eight miRNAs was validated using PerfeCTa miRNA primers (Quanta Biosciences, Gaithersburg, MD). In addition four miRNAs (miR-10, miR-15b, miR-16 and miR-24) were assessed for their suitability as a stable reference gene. Briefly, 15 µL of extracted RNA were reverse transcribed into cDNA using the NCode miRNA First-Strand cDNA Synthesis kit (Life Technologies, Carlsbad, CA). RT-qPCR was performed as previously described [4].

### Statistical analyses

Mann-Whitney rank sum test was used in analysing differences between the CFS/ME patients and non-fatigued controls regarding age and haematological characteristics (**Table 1** **and** **2**). For RT-qPCR analysis unpaired groups of values were compared according to the non-parametric Mann-Whitney U test. These data were analysed using GraphPad Prism 5 (GraphPad Software, San Diego, CA). Differential expression of the miRNA-Seq raw count data was assessed using the BioC/R package DESeq [29]. Due to the small sample size and the heterogeneity of the CFS/ME phenotype we interpreted significance from the unadjusted *P*-value, without the Benjamini-Hochberg method for False Discovery (FDR) correction. Statistical significance was accepted at *P*<0.05.

**Table 1:** Characteristics of CFS/ME and non-fatigued control participants.

Parameters	Non-Fatigued (n = 20)	CFS/ME (n = 20)	* *P*-values
**Age**	47.3±6.7	44.5±6.0	0.24
**White Blood Cells (10^3^/µL)**	6.19±1.58	5.53±1.45	0.12
**Lymphocytes (%)**	35.89±8.81	34.80±6.41	0.53
**Monocytes (%)**	6.72±1.60	6.96±1.77	0.66
**Granulocytes (%)**	57.39±8.94	58.25±6.49	0.74
**Lymphocytes (x10^3^/µL)**	2.14±0.53	1.89±0.46	0.13
**Monocytes (x10^3^/µL)**	0.41±0.12	0.39±0.15	0.66
**Granulocytes (x10^3^/µL)**	3.64±1.53	3.25±1.05	0.36
**Red Blood Cells (x10^6^/µL)**	4.20±0.36	4.13±0.24	0.43
**Haemoglobin (g/L)**	133.68±8.52	132.20±7.88	0.40
**Haematocrit (%)**	37.14±1.95	36.67±2.10	0.40
**Mean Cell Volume (fl)**	88.82±5.39	88.69±2.58	0.51
**Mean Cell Haemoglobin (pg)**	31.97±2.01	32.02±1.13	0.78
**Mean Corpuscular Haemoglobin Concentration (g/L)**	359.95±7.17	360.95±6.44	0.64
**Red Blood Cell Distribution Width (%)**	12.52±1.28	12.44±0.69	0.41
**Platelet (x10^3^/µL)**	231.06±88.61	243.80±53.20	0.38
**Mean Platelet Volume (fl)**	7.59±0.77	7.63±1.16	0.72

*Denotes statistical significance set at *P*<0.05.

The blood characteristics of the participants in the study are presented below following full blood count analysis.

**Table 2:** Characteristics of miRNA-Seq subset of CFS/ME and non-fatigued control participants.

Parameters	Non-Fatigued (n = 6)	CFS/ME (n = 6)	* *P*-values
**Age**	48.8±8.0	41.7±4.8	0.09
**White Blood Cells (10^3^/µL)**	6.23±0.83	5.67±1.60	0.70
**Lymphocytes (%)**	41.30±5.50	33.27±6.46	0.09
**Monocytes (%)**	7.02±1.20	6.42±1.22	0.49
**Granulocytes (%)**	51.68±5.28	60.32±6.02	**0.04** *
**Lymphocytes (x10^3^/µL)**	2.58±0.53	1.83±0.48	**0.03** *
**Monocytes (x10^3^/µL)**	0.43±0.08	0.37±0.16	0.49
**Granulocytes (x10^3^/µL)**	3.22±0.49	3.45±1.09	0.39
**Red Blood Cells (x10^6^/µL)**	4.24±0.39	4.06±0.26	0.24
**Haemoglobin (g/L)**	134.67±9.69	132.33±8.76	0.49
**Haematocrit (%)**	37.35±2.09	36.47±2.32	0.49
**Mean Cell Volume (fl)**	88.25±3.95	88.87±2.15	0.94
**Mean Cell Haemoglobin (pg)**	31.78±0.95	32.30±0.80	0.24
**Mean Corpuscular Haemoglobin Concentration (g/L)**	360.33±8.41	363.33±0.37	0.49
**Red Blood Cell Distribution Width (%)**	12.03±0.47	12.62±0.37	**0.03** *
**Platelet (x10^3^/µL)**	248.17±85.41	237.17±41.39	0.70
**Mean Platelet Volume (fl)**	7.58±0.80	6.90±0.86	0.24

* Denotes statistical significance set at *P*<0.05.

The blood characteristics of the participants chosen for sequencing analysis.

## Results

### Subject characteristics

The age of the study participants was recorded and a full blood count performed on whole blood samples prior to plasma separation. There were no significant differences between age or full blood count values in the CFS/ME and control groups (Table 1). For sequencing, a subset of six CFS/ME and six non-fatigued control samples were selected. A significant difference in lymphocyte and granulocyte percentages, lymphocyte count and red blood cell distribution width between the CFS/ME and control groups was observed in these subsets (Table 2).

### Illumina high-throughput sequencing of plasma miRNA in CFS/ME

**Figure 1:**
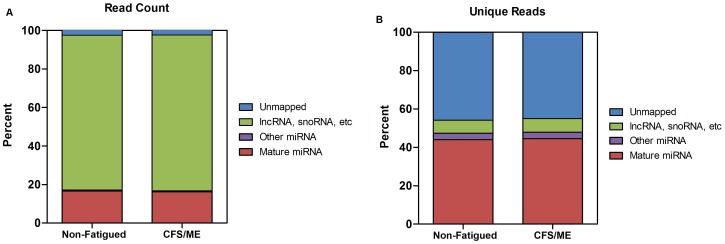
Read classification as predicted by miRanalyzer. Percentage of **A**) read count and **B**) unique reads mapped to mature miRNAs, other microRNAs (ambiguous, star and hairpin), other non-coding RNAs and unmapped.

To select for candidate plasma miRNAs differentially expressed in CFS/ME, we performed an initial genome-wide small RNA screening of six CFS/ME patients and six non-fatigued controls by Illumina HTS. A total of 154,989,205 reads were generated from a single flow cell lane on the HiSeq2000 sequencer. After demultiplexing an average of 12 million reads per library was obtained. Initial analysis of the library read lengths demonstrated a bimodal distribution of reads with peaks at 22 and 32 nucleotides (Figure S1). These populations were subsequently found to contain miRNA and long non-coding RNA (lncRNA) sequences respectively. On average 57.4% of decoded sequences were mappable, with 97.5% of mappable reads aligning to the human genome (hg18). Mature miRNA constituted 17% of all mapped reads, whilst other RNA species (including lncRNA and snoRNA) constituted 80.5% of mapped reads (Figure 1A). 75.1% of the mapped other RNA species consisted of lncRNA known as Ro-associated RNA Y4 (RNY4) which is the main component of the 32–33 nucleotide population. When examining unique reads, miRNA comprised 44.3% of all unique reads mapped whilst other RNA species comprised only 7% (Figure 1B).

### MiRNA characterisation

A total of 375 mature miRNA were initially identified in one or more samples based on sequence alignment to the miRBase registry (release 19). The top 25 expressed miRNAs in each sample group are listed in Table 3. Furthermore, 13 novel candidate miRNAs were predicted, although these amounted to a very small fraction of the total read count. However, none of these novel miRNA showed significant differences between CFS/ME and non-fatigued controls (Table S1).

**Table 3:** Top 25 most abundant miRNAs identified.

miRNA ID	Rank (Non-Fatigued)	Base Mean^1^ (Non-Fatigued)	Rank (CFS/ME)	Base Mean^1^ (CFS/ME)
**hsa-miR-486-5p**	1	232,025	1	206,637
**hsa-miR-191-5p**	2	163,282	2	161,507
**hsa-miR-92a-3p**	3	122,247	3	112,091
**hsa-miR-22-3p**	4	98,273	4	102,010
**hsa-miR-30d-5p**	5	64,038	5	58,586
**hsa-miR-26a-5p**	6	56,084	6	54,753
**hsa-miR-181a-5p**	7	49,098	7	37,346
**hsa-miR-151a-3p**	8	35,388	8	33,991
**hsa-miR-423-5p**	9	34,660	9	33,841
**hsa-miR-16-5p**	10	32,226	10	27,914
**hsa-miR-150-5p**	11	20,792	12	16,977
**hsa-miR-126-5p**	12	17,096	11	17,960
**hsa-miR-25-3p**	13	12,834	16	10,555
**hsa-miR-28-3p**	14	12,733	14	13,057
**hsa-miR-30e-5p**	15	12,559	13	13,146
**hsa-miR-186-5p**	16	11,374	17	10,153
**hsa-miR-151a-5p**	17	9,241	18	8,655
**hsa-miR-127-3p**	18	8,750	15	12,961
**hsa-miR-10a-5p**	19	8,093	22	6,443
**hsa-miR-423-3p**	20	7,619	19	7,856
**hsa-miR-451a**	21	6,451	23	6,292
**hsa-miR-140-3p**	22	6,252	25	5,895
**hsa-miR-27b-3p**	23	6,093	20	7,497
**hsa-miR-146a-5p**	24	5,994	21	7,246
**hsa-miR-221-3p**	25	5,749	29	4,863

1 The base mean is the mean of the counts for each miRNA divided by the size factor for each condition (as calculated by DESeq).

### Differential expression of plasma miRNA in CFS

Differential expression of identified miRNAs from miRBase was calculated using DESeq. A total of 19 microRNAs were significantly dysregulated in CFS/ME compared to non-fatigued controls (Table 4). Of the 19 differentially expressed miRNAs 16 were considered low in abundance due to a base mean count of less than 1,000 reads, their detection was found to be unreliable for confirmative RT-qPCR.The remaining three miRNAs (*hsa-miR127-3p*, *hsa-miR-142-5p* and *hsa-miR-143-3p*) were all up-regulated in CFS/ME compared to non-fatigued controls.

**Table 4:** miRNAs differentially expressed between CFS/ME and non-fatigued controls.

miRNA ID	Base Mean^1^ (Non-Fatigued)	Base Mean^1^ (CFS/ME)	Fold Change	*P*-value
**High-Abundance miRNAs**				
**hsa-miR-127-3p**	8,751.91	12,978.10	1.48	0.0476
**hsa-miR-143-3p**	2,060.61	4,439.17	2.15	0.0005
**hsa-miR-142-5p**	994.22	1,811.23	1.82	0.0443
**Low-Abundance miRNAs**				
**hsa-miR-331-3p**	366.94	590.85	1.61	0.0277
**hsa-miR-381-3p**	347.35	550.72	1.59	0.0336
**hsa-miR-136-3p**	125.63	224.69	1.79	0.0213
**hsa-miR-370**	59.84	130.44	2.18	0.0051
**hsa-miR-493-5p**	39.87	82.49	2.07	0.0263
**hsa-miR-4532**	10.47	78.06	7.45	0.0002
**hsa-miR-450b-5p**	1.72	1.23	0.71	0.0208
**hsa-miR-26a-1-3p**	1.56	1.16	0.75	0.0106
hsa-mir-126*	1.39	0.91	0.65	0.0449
**hsa-miR-5187-3p**	1.31	1.10	0.84	0.0217
**hsa-miR-641**	1.07	0.78	0.73	0.0240
**hsa-miR-548j**	1.07	1.10	1.03	0.0446
**hsa-miR-3065-3p**	0.90	0.71	0.79	0.0126
**hsa-miR-16-2-3p**	0.82	0.71	0.87	0.0128
**hsa-let-7g-3p**	0.82	0.78	0.95	0.0251
**hsa-miR-548ax**	0.82	0.91	1.11	0.0484

1 The base mean is the mean of the counts for each miRNA divided by the size factor for each condition (as calculated by DESeq). *The complementary analog for miR-126.

### RT-qPCR confirmation of plasma miRNA-Seq data

**Figure 2:**
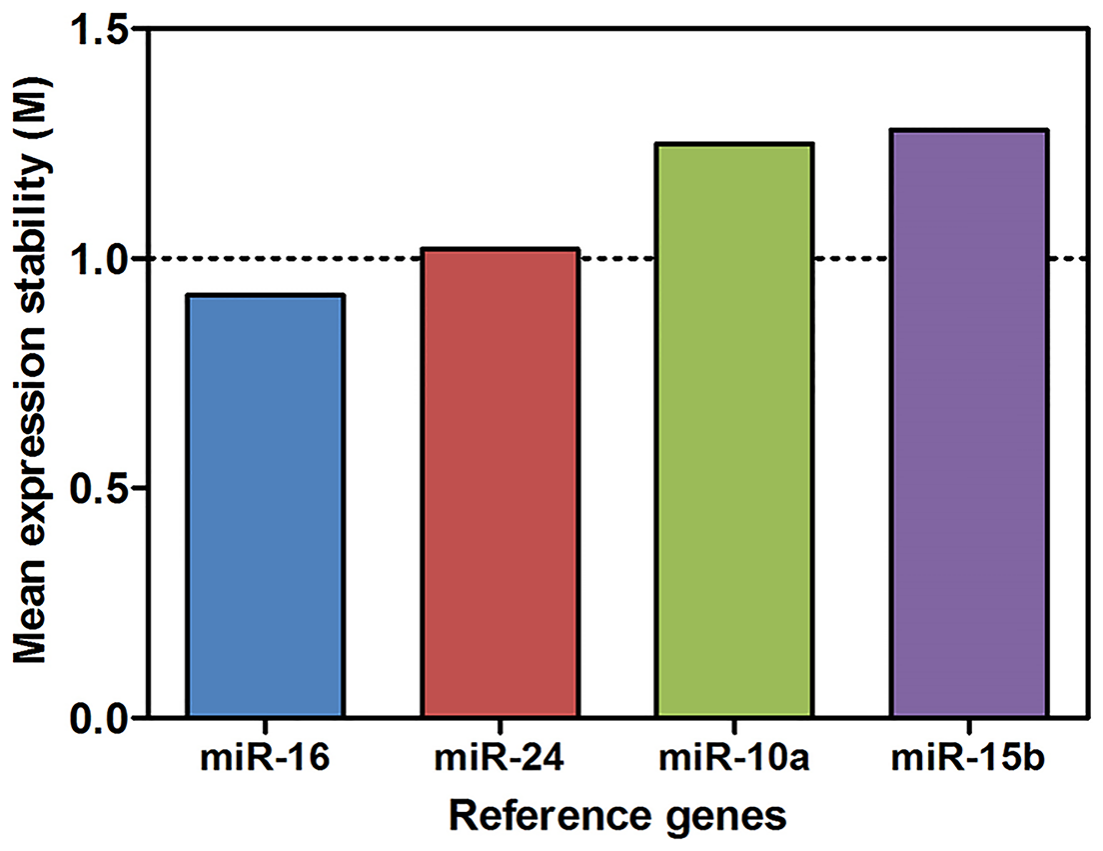
Expression stability values (M) of the putative reference genes tested in plasma. The expression of four genes was analysed to determine the most suitable reference gene. MicroRNAs are ranked from the most stable to the least stable (left to right). The dotted line indicates the recommended threshold value of <1.0 for heterogeneous samples.

**Figure 3:**
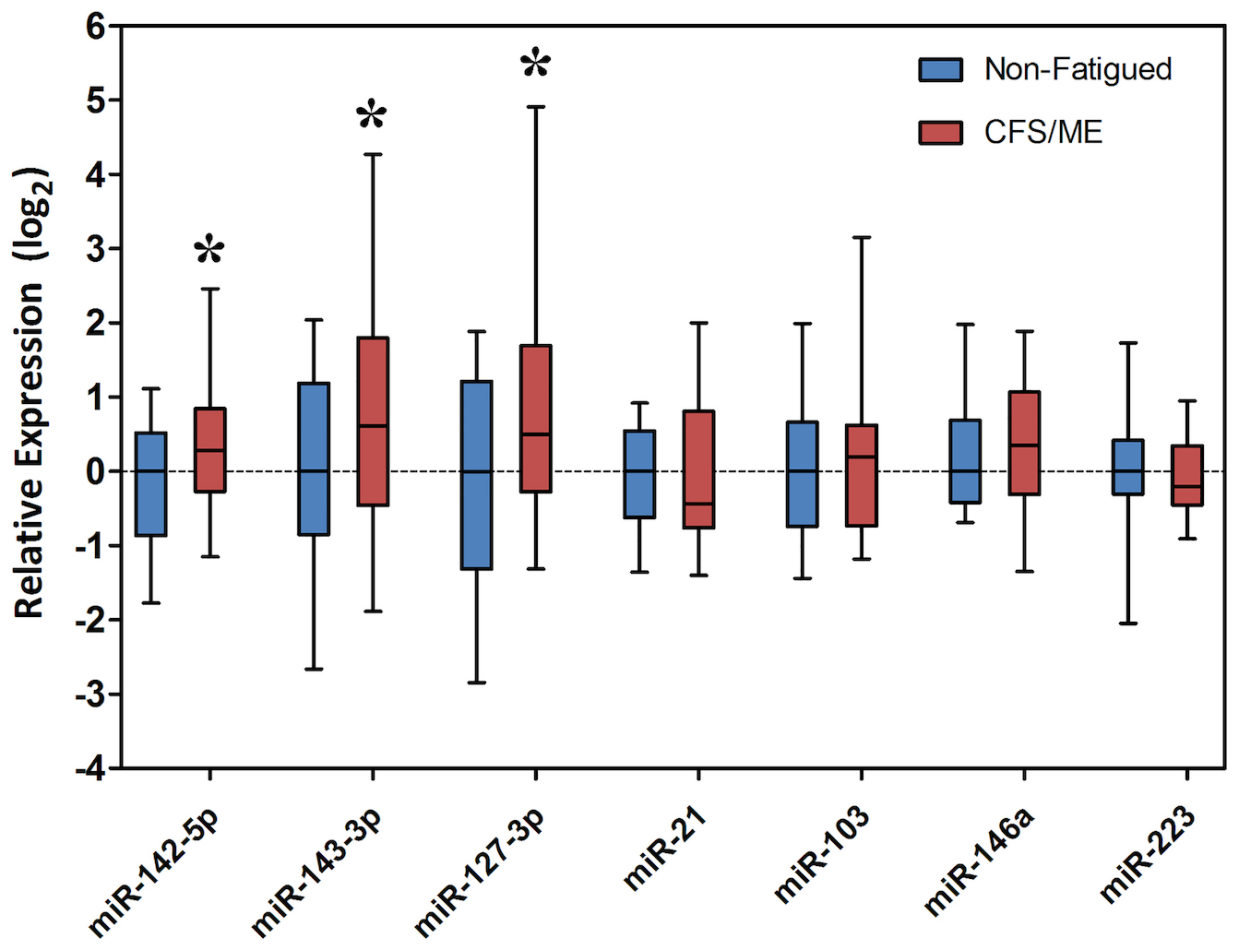
Relative expression data presented as boxplots for miRNAs identified as differentially expressed by Illumina HTS. Boxes indicate the interquartile range (25%–75%) with the horizontal bar within each box indicating the media. The whiskers show the minimum and maximum values. *P<0.05 vs. non-fatigued control (n = 20/group).

The choice of a stable reference gene is critical for accurate gene expression analysis by RT-qPCR. Of the four putative reference genes tested *hsa-miR-16-5p* was determined to be the most stably expressed (Figure 2). To validate the RNA-Seq results (n = 6/group) we performed RT-qPCR in an expanded sample cohort (n = 20/group). Three differentially expressed miRNAs (*hsa-miR127-3p, hsa-miR-142-5p and hsa-miR-143-3p*) and four non-differentially expressed miRNAs (*hsa-miR-21-5p, hsa-miR-103-3p, hsa-miR-146a-5p and hsa-miR-223-3p*) were selected and their expression levels quantified using RT-qPCR. The four non-differentially expressed miRNAs were selected as they previously demonstrated dysregulation in cytotoxic lymphocytes in CFS/ME patients [3], although HTS did not identify differential expression in plasma. All RT-qPCR results for the remaining three miRNAs were consistent with the RNA-Seq data (Figure 3).

## Discussion

To date, screening for CFS/ME has been based on well-established case definitions [3], [30]. Profiling of circulating miRNA levels may serve to enhance the molecular diagnosis of CFS/ME. Using Illumina HTS, the present study identified 19 miRNAs that were differentially expressed in CFS/ME patients. Of these, only three were confirmed to be highly abundant in the CFS/ME patients in comparison to the controls. These results suggest that *miR-127-3p, miR-142-5p* and *miR-143-3p* may be implicated in the pathogenesis of CFS/ME.

Although, there is currently no definitive source identified for the presence of miRNAs in biofluids, blood cells in particular reticulocytes, myeloid cells, lymphoid cells, platelets, cells from the liver, lungs and kidneys or lysed cells may release miRNAs into the circulation [31], . Similarly, miRNAs may be discharged into the plasma following tissue damage for example, circulating *miR-1* and *miR-133a*, are significantly increased following acute myocardial infarction [34]. The miRNAs in the present study have expressions in various tissues. *MiR-127-3p* is found in the testicular and nervous system [35], *miR-143-3p* is expressed in the colon [36] while *miR-142-5p* is expressed by cells of the immune system [37], [38]. However, both *miR-142-5p* and *miR-143-3p* are reported to be amongst the miRNAs frequently found in plasma and serum [32]. Over-expression of *miR-142-5p* has been observed in most cancer-related and immunological disorders [39]. This particular miRNA is abundant in most hematopoietic cell lines and may be involved in thwarting inflammatory processes [40]. In Systemic Lupus Erythematosus (SLE), increased expression of *miR-142-5p* in CD4^+^T cells prevents autoimmunity while a downregulation may result in autoreactive T cells and hyperactive B cells [41].

*MiR-142-5p* is important for T cell development where it targets SLAM associate protein (SAP). Inhibition of *miR-142-5p* may increase the expression of CD84, IL-10, SAP and IgG production [41]. CD84 is an important T cell regulatory marker as it regulates cytokine production, function, adhesion and interaction with B cells [42]. The levels of IL-10 have been shown to be equivocal in CFS/ME patients. The cause of an increase in *miR-142-5p* is unknown, however, it is likely that this may be related to heightened Treg suppression and additional autoimmune responses.

*MiR-143-3p* targets IgG Fcγ receptor 1 and also CD64 reducing lung inflammation. It is a tumour suppressor gene and is highly down regulated in colorectal cancer [43]. It inhibits the oncogene KRAS [44]. Overexpression of *miR-143-3p* in most cancer cells stagnates the growth of tumours and cancer cells [45] as it may act to reduce BCL2 mRNA thereby preventing tumour or cancer cell proliferation and promoting apoptosis [46]. *miR-143-3p* has been identified as a neutrophil specific miRNA [47]. Importantly, its expression is upregulated in cases of heightened erythropoiesis such as in polycythemia [48]. In CFS/ME increased levels of neutrophil apoptosis occurs in some patients [49], [50], [51], and this potentially ensues from high levels of *miR-143-3p*.

*MiR-127-3p* interferes with ERK signalling, a tumour suppressor and upregulations have been shown to increase apoptosis [52]. Importantly, it targets BCL6 a transcription factor which increases p53 expression [53]. BCL6 inhibits the production of IL-10 therefore by dampening BCL6 as a consequence of *miR-127* upregulation may result in significant increases in IL-10 [54]. In CFS/ME equivocal levels of IL-10 have been reported and an over expression of *miR-127-3p* may explain to some extent some of these patterns. BCL6 is an important transcription factor required for germinal centre B cell and follicular helper T cell development [55], [56], [57]. Irregularities in the expression of BCL6 may result in aberrant inflammatory responses and the development of various lymphomas [58].

The presence of a high proportion of the RNA Y4 within the small RNA sequencing libraries reduced miRNA sequencing capacity. Y RNAs are components of Ro ribonucleoproteins (RNPs) and were first identified in the serum of patients with the autoimmune disorder lupus erythematosus [59]. Y RNAs are similar in size and structure to miRNAs as they both have comparable stem and loop structures [60]. These similarities may explain the presence of Y RNA following small RNA library construction. Efficient depletion of Y RNA would yield higher quality HTS miRNA data allowing deeper sequencing. Methods for the reduction of Y RNA in a similar manner to rRNA depletion could be employed to improve the ratio of useful miRNA data [61]. Most extracellular miRNA studies do not report on the abundance of Y RNAs in circulation and this may be related to the read size filtering used. Given that Y RNAs upon degradation produce two classes of fragments and majority of which bind to Ro60, it is possible to posit that these fragments that we observed in the plasma samples are those Y RNAs bound to Ro60.

Plasma miRNAs show great promise as potential non-invasive biomarkers, but at present the precise and accurate measurement is challenging. A number of factors including cellular contamination, haemolysis and low quantity can result in significant bias that does not reflect the original biological state of the sample. Current circulating miRNA research indicates that haemolysis may influence the availability of miRNAs in circulation [33]. Haemolysis may be evaluated in archival data by examining the delta Cq of *miR-451* and *miR-23a* [33]. In healthy individuals, 194 miRNAs may be detected in both haemolysed and non-haemolysed blood samples, where 40.2% may be upregulated following haemolysis, 13% may be down regulated and 28.9% are unaffected by haemolysis [62], [63]. In the present study, our three candidate miRNAs, *miR-127-3p*, *miR-143-3p* and *miR-142-5p* are among the miRNA genes unaffected by haemolysis in healthy individuals [62].

The benefit of circulating miRNAs as biomarkers for diseases relates to a number of characteristics such as reduced complexity compared to proteins, stability, conserved sequences in various species and restricted expression in specific tissues and biological processes. It has been suggested that 206 miRNAs are expressed in blood cells, serum and plasma, thus in most plasma and serum studies it is highly necessary to observe strict sample processing procedures to ensure the samples are cell free [64]. Cellular content contamination of biofluids may be reduced by employing additional centrifugation steps during the initial plasma separation to lessen potential contamination from cellular debris and haemolysis [65]. Additionally, it may be important to assess blood cell counts and lysis during sample collection as variation in plasma levels of miRNAs in some cases have been credited to circulating blood cell effects [66], [67]. Starting concentration of miRNA levels vary among individuals due to age, sex and other factors and this may significantly impact the outcomes of various expression studies [68], [69]. To date there are no established reference values for miRNAs among normal individuals and this may be necessary for diagnostic marker purposes, hence, the inclusion of appropriate calibrator controls during RT-qPCR analysis is necessary. Nonetheless, despite these challenges, miRNA signatures from normal individuals are reproducible with similar expression patterns and a limited amount of variability [32], [70].

## Conclusions

The present study suggests that plasma may be a satisfactory parameter for determining miRNAs as biomarkers in CFS/ME. The data illustrates that *miR-127-3p*, *miR-142-5p* and *miR-143-3p* may be potential plasma biomarkers for CFS/ME diagnosis. However, further studies are now required to validate these findings in a larger cohort to ascertain diagnostic power of these findings. The shortcomings with using biofluid is the low abundance of RNA thus a large quantity of sample is often required to achieve high yields and quality of RNA. Presently, the use of HTS as a means to detecting possible changes in miRNAs in disease presentations is in its infancy. Further studies are required to evaluate and refine the method to promote better detection and reliable data that can be replicated across studies.

## Supporting Information

Figure S1 **Length distribution of sequenced small RNA.** Data represents mean ± SEM (n = 6/group).
(TIF)

Table S1 **Novel miRNA candidates as predicted by miRanalyzer.**
(DOCX)
