# Supplementary figures and images for: High-Throughput Sequencing of Plasma MicroRNA in Chronic Fatigue Syndrome/Myalgic Encephalomyelitis
Source: PLoS One. 2014 Sep 19;9(9):e102783. doi: 10.1371/journal.pone.0102783 (PMC4169517; doi:10.1371/journal.pone.0102783)

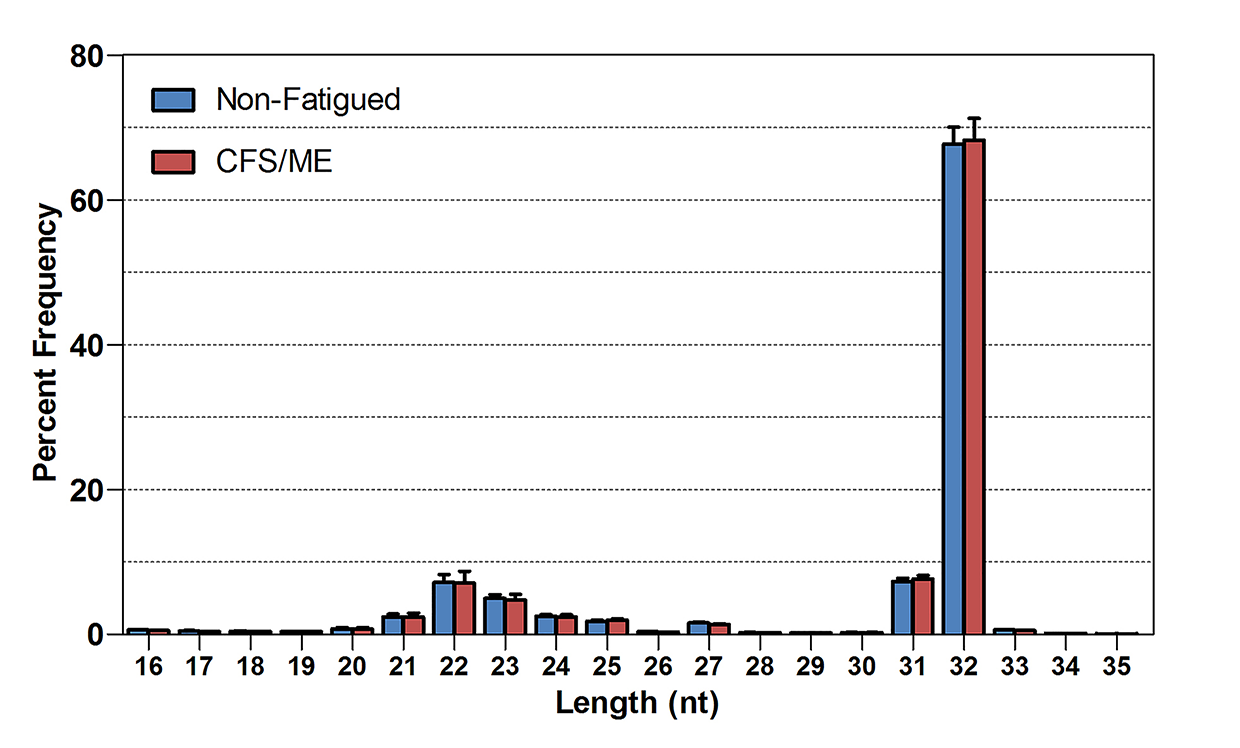

Supplement: Figure S1 — Length distribution of sequenced small RNA. Data represents mean ± SEM (n = 6/group). (TIF) [file pone.0102783.s001.tif]
