# Supplementary material for: High-Throughput Sequencing of Plasma MicroRNA in Chronic Fatigue Syndrome/Myalgic Encephalomyelitis
Source: PLoS One. 2014 Sep 19;9(9):e102783. doi: 10.1371/journal.pone.0102783 (PMC4169517; doi:10.1371/journal.pone.0102783)
Supplement: Table S1 — Novel miRNA candidates as predicted by miRanalyzer. (DOCX) [file pone.0102783.s002.docx]

**Table S1:** Novel miRNA candidates as predicted by miRanalyzer

| **miRNA Candidate** | **chrom** | **chromStart** | **chromEnd** | **Strand** | **consensusMature** | **consensusPrecursor** |
| --- | --- | --- | --- | --- | --- | --- |
| Candidate_1 | chr2 | 133,038,048 | 133,038,170 | - | AGGGCTGGGTCGGTTGGGCTGGG | ATCCATAACTTCGGGATAAGTATTGGCTGTAAGGGCTGGGTCGGTTGGGCTGGGTGCGTGCCGCCGCTCGACGAGGCCCCGCTGCCCCCCCCAAGCCCCAGGAATGCCTGTTGTGGCCCTCCC |
| Candidate_2 | chr2 | 64,567,878 | 64,567,986 | - | ACAGGAGTGGGGGGTGGGACGT | ATCCTGTGTTCCCTATCCTCCTTATGTCCCACCCCCACTCCTGTTTGAATATTTCACCAGAAACAGGAGTGGGGGGTGGGACGTAAGGAGGATGGGGGAAAGAACATCA |
| Candidate_3 | chr2 | 64,567,877 | 64,567,989 | - | TATGTCCCACCCCCACTCCTGTTT | TTCATCCTGTGTTCCCTATCCTCCTTATGTCCCACCCCCACTCCTGTTTGAATATTTCACCAGAAACAGGAGTGGGGGGTGGGACGTAAGGAGGATGGGGGAAAGAACATCAT |
| Candidate_4 | chr19 | 58,024,354 | 58,024,452 | - | TCCCTGTTCGGGCGCCA | CTCCAGCAGACCTTCTTCACTCACGTCCCTGTTCGGGCGCCACTTGTGGCTGTCGGTTCGGGACTGAATGAAGAAGGACAAATGCAGAAATGAAGACAA |
| Candidate_5 | chr16 | 81,902,006 | 81,902,142 | - | GGACGGGCTTGGCAGAATCAG | CTACTGACTCTACCTGCCACCCAGAGGAACCAAAACCACAGGACGGGCTTGGCAGAATCAGCCAGGAAAGACCCAGCTGAATTTGACTCTAGTCTGACACTGTGAAGAGACAAGAGGGATGCAGAGTAAGTGGGGAC |
| Candidate_6 | chr22 | 43,011,306 | 43,011,426 | + | GATGCCTGGGAGTTGCGATCTGC | ACTGCTAATGTGAGACGAATTTTTGAGCGGGTAAAGGTCGCCCTCAAGGTGACCCGCCTACTTTGCGGGATGCCTGGGAGTTGCGATCTGCCCGACCTTATTCACGCCTAAAAAGTAGACT |
| Candidate_7 | chr20 | 57,598,899 | 57,598,989 | + | CTCCGGGATGGGCACTCTGCTCA | GGGCGGGGGCACAGGCTCCGGGATGGGCACTCTGCTCATGAACAAGATTAGAGAGGAGTACCCGGACCGGATCATGAATTCCTTCAGCGTC |
| Candidate_8 | chr8 | 22,102,453 | 22,102,581 | - | AAAAGCTGGGTTGAGAGGGCGA | TGGGCCACAGTATTTATCAGGCGGCGCTTCGCTCCCCTCCGCCTTCTCTTCCCGGTTCTTCCCGGAGTCGGGAAAAGCTGGGTTGAGAGGGCGAAAAAGGATGAGGTGACTGGTCTGGGCTACGCTATG |
| Candidate_9 | chr7 | 148,638,568 | 148,638,668 | + | CGAGTGTTGTGGGTTATTG | ACAGGATAACACAGTTGGTCCGAGTGTTGTGGGTTATTGTTAAGTTGATTTAACATTGTCTCCCCCCACAACCGCGCTTGACTAGCTTGCTGTTTTGCACT |
| Candidate_10 | chr7 | 143,079,609 | 143,079,705 | + | CTCTGACCTCTGACCCTCTAG | TTACCTCCCCTGCACCTCTGCCTTGGGGGTGGGGGGATAGAGGCATGGAATAGGTGCTCTGACCTCTGACCCTCTAGCCCAGGGAGAAGGTGAGCAG |
| Candidate_11 | chr6 | 5,831,634 | 5,831,730 | - | ACCACACCCACTGGAGAACTC | CAGCCCACGGCAGCCAGAGAACCACACCCACTGGAGAACTCTAGGTTCTCTACGGCCTCTTGTTACTGCCAGTGCTTGTGTGGTTTGAGGTGGGTAA |
| Candidate_12 | chr4 | 147,329,720 | 147,329,860 | + | GACAATTGTTGATCTTGGGCCTG | ATGGAAAGAGCTTGAGACTTTATATTGTTGAACATCAGACTCAAGGCCAACAACTGACTACTTCGAAAATGGGAAGTAGACAATTGTTGATCTTGGGCCTGATGTTCAACAATATAAAGTTGACTGGTGGACACTAATGGA |
| Candidate_13 | chr3 | 49,058,041 | 49,058,149 | + | GGACGAAATCCAAGCGCAGCTG | GGCGCTGCTCAGGCAGGAGAGCAGGGGACGAAATCCAAGCGCAGCTGGAATGCTCTGGAGACAACAGCTGCTTTTGGGATTCCGTTGCCCGCTGTCCAGCCGTTGGCGG |
